# The association between subjective caregiver burden and depressive symptoms in carers of older relatives: A systematic review and meta-analysis

**DOI:** 10.1371/journal.pone.0217648

**Published:** 2019-05-29

**Authors:** Rafael del-Pino-Casado, Marta Rodríguez Cardosa, Catalina López-Martínez, Vasiliki Orgeta

**Affiliations:** 1 Department of Nursing, School of Health Sciences, University of Jaén, Jaén, Spain; 2 Division of Psychiatry, Faculty of Brain Sciences, University College London, United Kingdom; University of Mississippi Medical Center, UNITED STATES

## Abstract

**Background:**

Family carers are an important source of care for older people. Although several studies have reported that subjective caregiver burden is related to depressive symptoms there are no systematic reviews quantifying this association.

**Objective:**

To establish the extent to which subjective caregiver burden is associated with depressive symptoms and whether this association would vary by study or care characteristics.

**Methods:**

We searched major databases such as PubMed, CINAHL, PsycINFO, Scopus and ISI Proceedings up to March 2018, and conducted a meta-analysis of included studies. Summary estimates of the association were obtained using a random-effects model to improve generalisation of findings.

**Results:**

After screening of 4,688 articles, 55 studies were included providing a total of 56 independent comparisons with a total of 9,847 carers from data across 20 countries. There was a large, positive association between subjective caregiver burden and depressive symptoms ($\bar{r}$ = 0.514; 95% CI = 0.486, 0.541), with very low heterogeneity amongst individual studies (I^2^ = 8.6%). Sensitivity analyses showed no differences between cross-sectional or repeated measures ($\bar{r}$ = 0.521; 95% CI = 0.491, 0.550; 51 samples) and longitudinal studies ($\bar{r}$ = 0.454; 95% CI = 0.398, 0.508; 6 samples). We found a higher effect size for those caring for people living with dementia compared to those caring for frail older people, and stroke survivors. Carer sex, age and kinship did not change the estimate of the effect.

**Conclusions:**

Subjective caregiver burden is a significant risk factor for depressive symptoms in carers of older people and may precipitate clinical depression. Those caring for people with dementia experience greater burden. There is a need for longitudinal evaluations examining the effects of potential mediators of the association of subjective burden and depressive symptoms. Future interventions should test whether minimizing subjective burden may modify the risk of developing depression in carers of older relatives.

## Introduction

Current trends in population aging in many countries mean that as the population of older people increases so does the need for provision of informal care by family members [1]. Increases in age-related morbidity and disability increase old age dependency which is projected to double by 2050 [2]. In fact family carers are the main source of support of older dependents [1]. Although this uncompensated support is an important societal asset, it is associated with substantial health burden for family carers representing a highly vulnerable population [3].

Caregiving is associated with negative consequences for family carers’ physical and mental health [3]. The emotional and psychological consequences of caring are mainly represented by subjective burden, anxiety and depressive symptoms [4–6]. Prevalence studies have shown that depressive symptoms in carers of older relatives is 40.2% for those caring for stroke survivors [5] and up to 34% for carers of people living with Alzheimer’s disease [6].

Theoretical models explaining the negative emotional consequences of caregiving have been largely based on Lazarus and Folkman’s Transactional Stress process model [7]. According to this model, stress consequences are mediated by the way carers’ perceive, evaluate and manage the caregiving process [8]. In this context, subjective caregiver burden is defined as a caregiving state, characterised by a negative reaction to the impact of providing care [9], whereby vulnerability to burden is due to several factors such as carers’ physical health, psychological well-being, finances, social support and relationship with the care-recipient [10]. Objective burden is considered to reflect daily and practical aspects of provision of care capturing quantitative dimensions of the caregiving role such as level of care needs and hours providing care [11].

Several studies have been conducted to explore the possible association of subjective burden and depressive symptoms in carers of older people [12–14]. To date, systematic reviews in the area have included only cross-sectional studies [14], or have provided a narrative [12,13] as opposed to a quantitative synthesis of the literature. In addition, no review has commented on the methodological quality of the evidence, or assessed for effects of publication bias or conducted sensitivity analyses of factors influencing this association. Consequently a meta-analysis that quantifies the effect of the association whilst also reporting on the quality of the evidence is very much needed. In this paper we describe a systematic review and meta-analysis of the published literature to date reporting on the association of subjective caregiver burden and depressive symptoms and comment on the quality of the evidence.

The objectives of the present review were to establish the extent to which subjective caregiver burden is associated with depressive symptoms and whether this association would vary by study design, methodological quality, carer or care recipient characteristics.

## Material and methods

### Design

We followed published guidelines on methodology of reviews [15], Cochrane Handbook guidelines [16] and reported findings using the PRISMA [17] and MOOSE statements [18].

### Search strategy and selection criteria

Electronic databases (PubMed, CINAHL–EBSCO-, PsycINFO–ProQuest-, Scopus–Elsevier- and ISI Proceedings) were searched without time or language limits. We used search terms such as caregivers (MeSH term) or carer(s); burden, strain or role overload and depression (MeSH term), depressive symptom(s), depression or depressive (see S1 Appendix), up until March 2018. We conducted manual searches of relevant scientific journals (nursing, psychological and medical) and searched reference lists of included papers and reviews in the area [4,19,20] from January 1990 to March 2018.

Studies were included if they met the following criteria: (a) reported on an original quantitative investigation about informal carers of older care-recipients (≥ 65 years or more), (b) examined the association between subjective caregiver burden and depressive symptoms and (c) reported a correlation coefficient or another statistical metric that allowed calculation of a correlation coefficient.

To increase the validity of our eligibility criteria, we defined as “informal carers” someone who provided unpaid care (family members, friends, community members or volunteers) and those who cared both at home and in institutions [21]. We considered an “older care-recipient” any person over 65 years of age who scored as dependent in at least one activity of daily living (or instrumental activity of daily living). Depressive symptoms were defined as sad mood, loss of interest or joy in daily activities, fatigue, and excessive feelings of guilt and worthlessness [22,23]. In all studies subjective burden was defined as a caregiving state, reflecting the emotional, psychosocial and physical aspects of the caregiving role [10,11] measured by burden specific scales (i.e. the Zarit Burden Interview, Screen for Caregiver Burden, Caregiver Burden Inventory etc). Studies differed in the way they defined objective burden; this was measured by self-report measures of duration and/or hours of providing care, level of cognitive and/or functional impairment of the care recipient, disease severity or burden related to disease-specific symptoms.

Selection of studies was independently conducted by two reviewers (RdPC and MRC; Kappa: 0.78) and disagreements were resolved by consensus (discussion and agreement among the two reviewers).

### Data extraction and synthesis

Two independent reviewers (RdPC and MRC) extracted data on sample characteristics, study design, effect estimates and quality criteria of each study using a standardised data extraction form (kappa: 0.79). Disagreements were resolved by consensus (discussion and agreement among the two reviewers). The effect size measure used to pool data was the correlation coefficient, adjusted by the inverse of the variance using a random effects model. We classified the effect size following Cohen’s criteria [24] as: 0.1–0.29 (small), 0.3–0.49 (moderate) and higher than 0.5 (large). In repeated measured studies with no relation between time points, the first measure was chosen.

### Quality assessment

Following the recommendations of Boyle [25] and Viswanathan et al. [26], we used the following criteria for assessing methodological quality of individual studies: (1) sampling: probabilistic sampling, (2) measurement: i) details of the measurement process, ii) content validity and internal consistency of measures in the target or similar population, and iii) absence of information bias; (3) control for confounding factors: at least one measure of objective burden must be controlled for and (4) adequate reporting of statistical analysis. Criteria 2 and 4 were considered mandatory for a study to be included in the meta-analysis.

Regarding control of confounders, objective burden was considered necessary given its association with depression [4]. Objective burden encompasses functional capacity, cognitive impairment and behavioural problems [27]. Because measures of previous dimensions of objective burden are highly intercorrelated [20], we decided to control for at least one of these. We considered as high quality any study that controlled for confounding via allocation between groups (e.g., through stratification or matching) or controlling for confounding variables in the design and/or analysis (e.g. through multivariate analysis) [26]. If statistical adjustment was reported, we considered no confounding bias to be present if variation of the point estimate was less than 10% [28]. Two independent reviewers assessed quality (RdPC and CLP) and any disagreements were resolved by discussion with a third reviewer.

Following the recommendations of Meader et al. [29], based on the Grading of Recommendations Assessment, Development and Evaluation (GRADE) [30], imprecision, inconsistency and risk of publication bias were also assessed. Imprecision was evaluated through: a) number of included studies (large: >10 studies, moderate: 5–10 studies and small: <5 studies) and b) median sample size (high: >300 participants, intermediate: 100–300 and low: <100). Inconsistency was measured by heterogeneity of findings in individual studies. Publication bias was assessed by a funnel plot and statistical tests.

### Analysis

Following recommendations of Cooper et al. [31], a random effects model was used for the meta-analysis in order to improve generalisation of findings. We further computed the relative risk reduction from the pooled correlation coefficient based on recommendations of Borenstein et al. [32] and Higgins and Green [33].

The Q test was used for quantifying heterogeneity alongside inconsistency (I^2^) [34]. We used several methods for evaluating publication bias (Guyatt et al. [35] such as a funnel plot, the Begg’s test [36], the Egger’s test [37] and the Trim and Fill method [38]. The Begg’s and Egger’s test evaluate asymmetry of the funnel plot with a p value less than 0.10 indicative of publication bias [31] whereas the Trim and Fill method computes the combined effect considering a possible publication bias [38].

We performed sensitivity analyses to assess the robustness of findings using the leave-one-out method and subgroup analyses. The leave-one-out method consists of performing k-1 meta-analyses removing one study and analysing the remaining k-1 studies each time. We used subgroup analyses and metaregression to analyse the influence of study design, methodological quality of individual studies, care recipient illness and carer characteristics (age [mean], sex [% of woman] and kinship [% of spouses]) on meta-analysis results. Analyses were carried out using Comprehensive Meta-Analysis 3.3 software.

## Results

**Fig 1:**
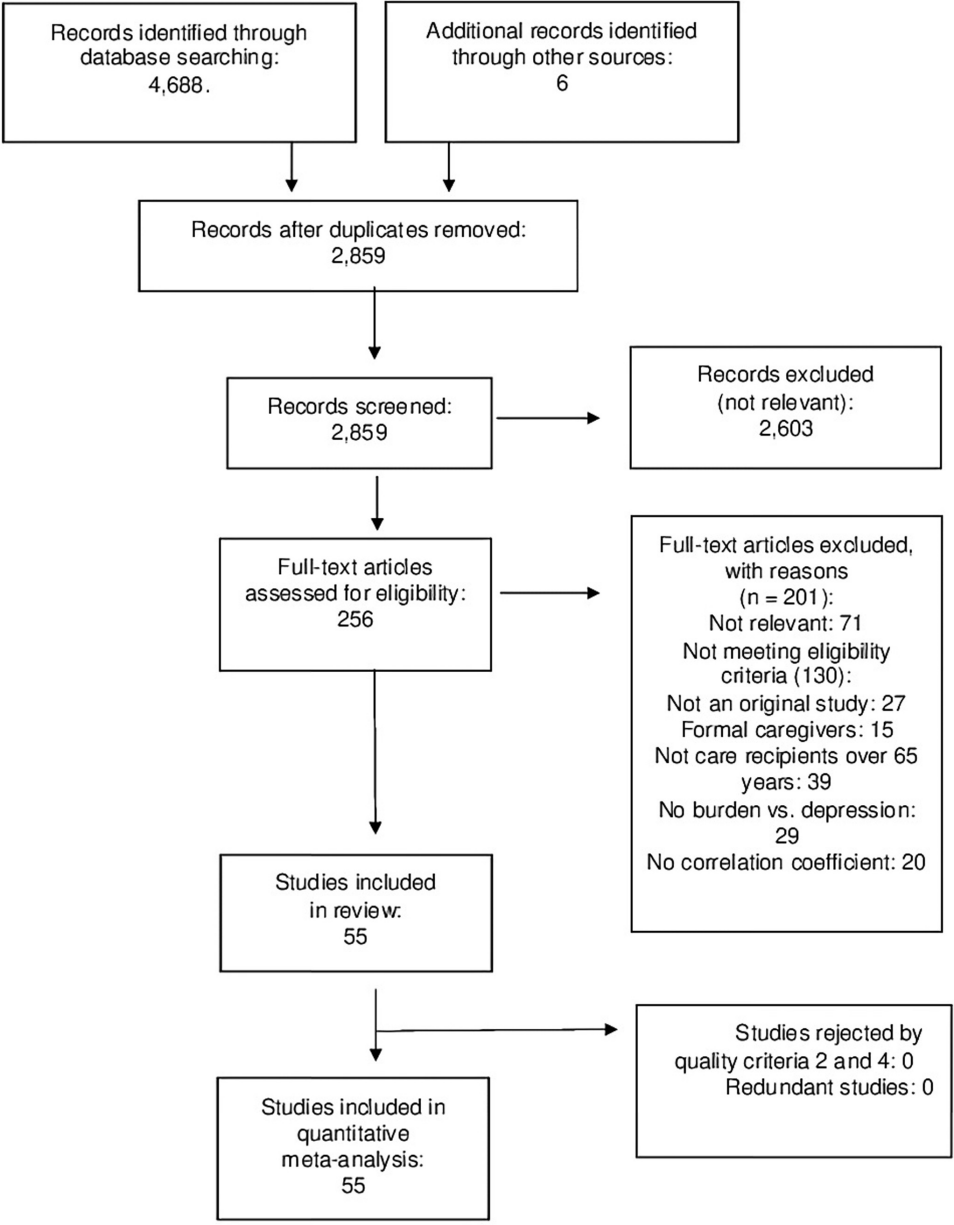
PRISMA flow diagram of the review process.

A total of 4,688 records were retrieved from searching databases and six further references were identified by manual search (Fig 1; Flow diagram of the search process). After removing duplicates, 2,859 records were screened, of which 2,603 were excluded as not relevant leaving 256 studies assessed for eligibility. Of these, 71 were excluded as not relevant and 130 not meeting inclusion criteria. We included a total of 55 studies all of which were assessed for quality and included in the meta-analysis [39–93]. All studies met both quality criterion 2 (measures) and 4 (adequate statistical analysis).

Characteristics of the 55 studies meeting inclusion criteria are presented in Table 1; there were 56 independent samples and 56 independent comparisons. Most studies were cross-sectional or repeated measures studies (with cross-sectional correlations) (n = 49); the majority (89%) reported on non-probabilistic samples (n = 47) and half of the studies did not report controlling for confounders (n = 28). The main care recipients were people with dementia (n = 31) and frail older people (n = 14). The included studies came from 20 different countries.

**Table 1:** Description and quality criteria of the studies included in the meta-analysis.

Author, year	N	Design	Care recipients	Probabilistic sampling	Appropriate measures	Control of confounders
Adams et al.2008	428	Cross-sectional	Dementia	-	+	+
Alspaugh et al. 1999	188	Longitudinal	Dementia	-	+	+
Ar 2017	190	Cross-sectional	Dementia	-	+	+
Bachner 2016	125	Cross-sectional	Cancer	-	+	?
Bianchi et al. 2016	121	Cross-sectional	Frail older people	-	+	+
Brandão et al. 2017	43	Cross-sectional	Frail older people	-	+	?
Buyn 2013	63	Repeated measures	Stroke	-	+	+
Carter et al. 2008	219	Cross-sectional	Dementia	-	+	+
Cheng et al. 2013	142	Cross-sectional	Dementia	-	+	-
Chow & Ho 2012	158	Cross-sectional	Frail older people	-	+	+
Clark et al 2013	106	Cross-sectional	Frail older people	-	+	+
Clyburn et al 2000	613	Cross-sectional	Dementia	+	+	-
Cooper et al. 2008	83	Cross-sectional	Dementia	-	+	+
Corazza et al. 2014	30	Cross-sectional	Dementia	-	+	?
D'Aoutst et al. 2014	53	Cross-sectional	Dementia	-	+	?
Del-Pino-Casado et al. 2015	200	Cross-sectional	Frail older people	+	+	+
Del-Pino-Casado et al. 2017	200	Cross-sectional	Frail older people	+	+	+
Diehl-Schmid et al. 2013	104	Cross-sectional	Dementia	-	+	-
Dos Santos et al. 2017	36	Cross-sectional	Mental illness	+	+	?
Drinka et al. 1987	127	Cross-sectional	Frail older people	-	+	+
Edelstein et al. 2017	107	Cross-sectional	Frail older people	-	+	?
Gallager et al. 2011	84	Cross-sectional	Dementia	-	+	+
González-Abraldes et al. 2013	33	Cross-sectional	Dementia	-	+	?
Graf et al. 2017	72	Longitudinal	Stroke	-	+	+
Grano et al. 2017	170	Longitudinal	Dementia	-	+	?
Heo & Koeske 2013	642	Cross-sectional	Dementia	-	+	?
Hirschman et al. 2004	251	Cross-sectional	Dementia	-	+	?
Jarazc et al. 2012	150	Cross-sectional	Stroke	-	+	+
Jones et al 2015	76	Cross-sectional	Cancer	-	+	?
Karabekiroğlu et al. 2018	69	Cross-sectional	Cancer	-	+	?
Khalaila & Litwin 2011	250	Cross-sectional	Frail older people	+	+	+
Kim et al. 2016	476	Cross-sectional	Dementia	-	+	?
Kowalska et al. 2017	58	Cross-sectional	Dementia	-	+	?
Kruithof et al 2016	183	Longitudinal	Stroke	-	+	+
Lai 2009	339	Cross-sectional	Frail older people	+	+	+
Lawton et al. 1991	285 (1)	Cross-sectional	Dementia	-	+	+
	244 (2)	Cross-sectional	Dementia	-	+	+
Li & Lewis 2013	65	Cross-sectional	Dementia	-	+	?
Liu et al. 2012	180	Cross-sectional	Dementia	-	+	?
Liu et al. 2017	120	Cross-sectional	Dementia	-	+	+
Lopez-Martínez et al. 2017	132	Cross-sectional	Frail older people	+	+	+
Luther 2014	150	Cross-sectional	Dementia	-	+	+
Mausbach et al. 2012	126	Cross-sectional	Dementia	-	+	-
McCullag et al. 2005	232	Longitudinal	Stroke	-	+	?
Medrano et al. 2014	67	Cross-sectional	Dementia	-	+	?
Mohamed et al. 2010	421	Cross-sectional	Dementia	-	+	+
Morlett Paredes 2014	103	Cross-sectional	Dementia	-	+	?
Parker 2007	40	Cross-sectional	Dementia	-	+	?
Powers 2014	83	Cross-sectional	Frail older people	-	+	+
Raveis et al. 1998	164	Cross-sectional	Cancer	-	+	?
Robison-Surgot & Knight 2005	48	Cross-sectional	Dementia	-	+	?
Romero Moreno et al. 2011	167	Cross-sectional	Dementia	-	+	+
Sutter et al. 2016	127	Cross-sectional	Dementia	-	+	?
Vitaliano et al. 1991	79	Longitudinal	Dementia	-	+	?
Wang et al. 2017	621	Cross-sectional	Frail older people	+	+	+
Yates et al. 1999	204	Cross-sectional	Frail older people	-	+	+

Notes: (+) characteristic is present; (-) characteristic is absent; (?) there is not enough information to assess.

Meta-analysis indicated a large, positive pooled effect ($\bar{r}$ = 0.513; 95% CI = 0.484, 0.541; N = 9,847; median sample size: 172.8) whereby high levels of subjective caregiver burden were associated with higher levels of depressive symptoms. The pooled effect is equivalent to an absolute risk reduction of 0.14; so if we eliminate or prevent subjective burden, risk of depressive symptoms would decrease by 14 percentage points.

**Fig 2:**
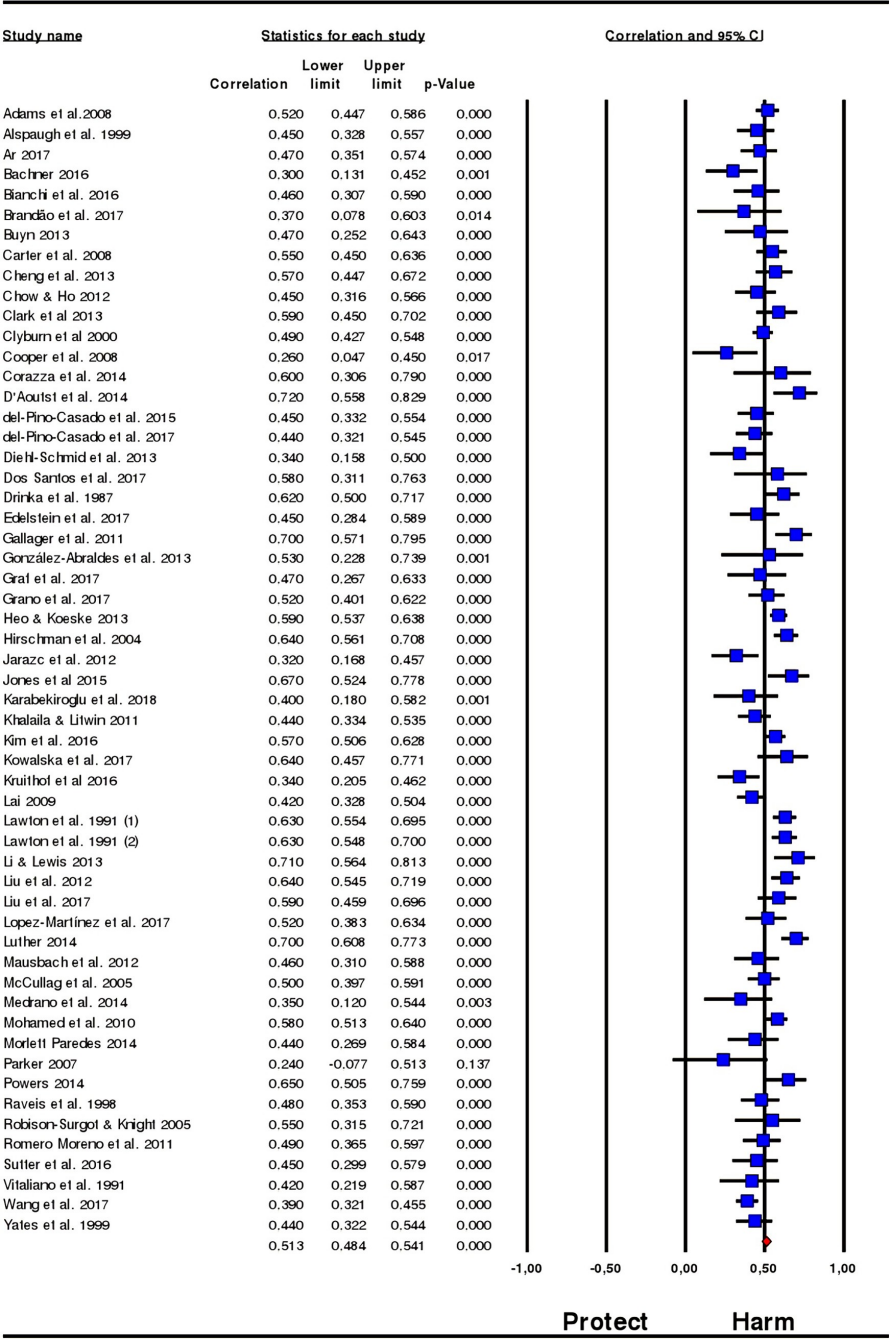
Forest plot for subjective caregiver burden and depressive symptoms.

**Fig 3:**
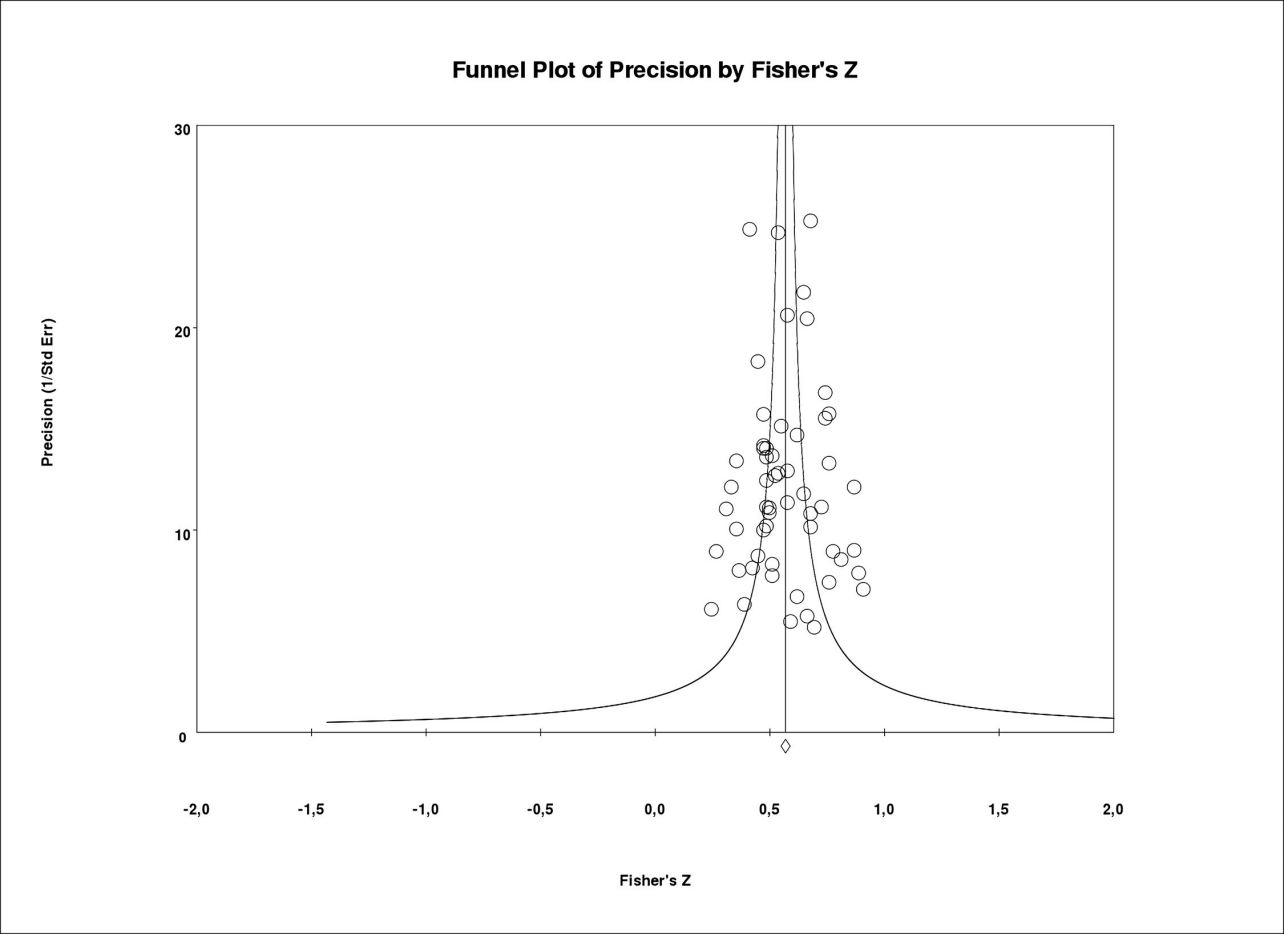
Funnel plot for subjective caregiver burden and depressive symptoms.

The correlation coefficient was positive in all individual samples except in one (Fig 2). The leave-one-out method yielded variations in the combined estimate under 0.7% (from 0.509 to 0.517). Because of the width of confidence intervals (CIs), the number of studies and the median sample size we can be confident that results are precise. There was very low heterogeneity amongst individual studies (Q = 60.19, degree of freedom [df] = 55, p = 0.29, I^2^ = 8.6%) and inspection of the funnel plot indicated that publication bias was not present (Fig 3). The Egger’s test (p = 0.92) and the Begg’s test (p = 0.98) confirmed this. Statistical power for these tests was 83% [94] and the pooled effect calculated by the Trim and Fill method showed no variation ($\bar{r}$ = 0.513).

Regarding quality criteria, no differences were found between studies controlling for objective primary stressors ($\bar{r}$ = 0.507; 95% CI = 0.467, 0.545; 26 samples) and those that did not ($\bar{r}$ = 0.519; 95% CI = 0.477, 0.559; 31 samples). There was an effect however regarding differences between studies with probabilistic samples ($\bar{r}$ = 0.446; 95% CI = 0.411, 0.479; 8 samples) and those without ($\bar{r}$ = 0.524; 95% CI = 0.492, 0.554; 48 samples).

Additional meta-analyses found no effect of type of design with no differences between cross-sectional or repeated measures ($\bar{r}$ = 0.520; 95% CI = 0.490, 0.550; 50 samples) versus longitudinal studies ($\bar{r}$ = 0.454; 95% CI = 0.398, 0.508; 6 samples). When examining care recipient illness we found higher effect sizes for those caring for people living with dementia compared to those caring for frail older people and stroke survivors (Table 2).

**Table 2:** Pooled effect of subjective caregiver burden on depressive symptoms by care recipient illness.

Care recipient	k	$\bar{r}$	95% CI of $\bar{r}$	P-value	I^2^	$\bar{r}$ corrected by Trim & Fill
Mental illness	1	0.580	0.311; 0.763	0.0001		
Dementia	32	0.547	0.513; 0.579	< 0.0001	24.8%	0.547
Cancer	4	0.471	0.305; 0.609	< 0.0001	9.8%	0.471
Frail older people	14	0.470	0.427; 0.511	< 0.0001	5.2%	0.472
Stroke	5	0.416	0.331; 0.494	< 0.0001	0.0%	0.416

Our metaregression showed that care recipient illness and type of sampling method accounted for 45% of heterogeneity. Sex (percentage of female; p = 0.80), age (mean; p = 0.97) and kinship (% of spouses; p = 0.30) of carers did not contribute to the regression model.

## Discussion

To our knowledge, this is the first systematic review and meta-analysis examining the association of subjective caregiver burden and depressive symptoms in informal carers of older people. By including all available evidence to date we found that experiencing subjective caregiver burden was associated with a moderate increased risk of depression. Our meta-analysis is an important contribution to the literature as it is the first to assess the methodological quality of studies and the influence of parameters such as characteristics of care recipients. Our analyses in fact included many studies across 20 countries and a total of 9,847 carers of older relatives. We found that the association between subjective caregiver burden and depressive symptoms represents a large effect. We can be confident that our findings are relatively robust given the low heterogeneity observed. Our conclusions can be further strengthened by the fact that effects were consistent across studies and there was no evidence of publication bias.

We have been able to include recent studies compared to previous meta-analyses [20] and provide an estimate of the effect. Regarding the methodological quality of research conducted to date we found limitations in the design, sampling methods and control of confounders. Given therefore limitations in the current literature, we can conclude that evidence to date is of moderate quality. We also report that type of sampling method influenced our results.

An important concern in systematic reviews of observational studies is controlling for the effect of confounders [26]. In the present study, we applied several strategies for addressing this issue and we found that controlling for levels of objective burden experienced by carers in individual studies did not influence the pooled estimate. Our findings are consistent with previous reviews [12–14] but additionally expand the evidence by demonstrating that the association of subjective caregiver burden and depressive symptoms is a robust one, based on moderate quality evidence, and generally represents a large effect. An important strength of our review is that studies were consistent in their definition and measurement of subjective caregiver burden as a psychological construct [95].

In previous reviews [12–14], most of the studies employed cross-sectional designs, which prevents conclusions about causality. In the present review, we included six longitudinal studies and have demonstrated no statistical differences between the pooled effect of cross-sectional versus longitudinal studies; our findings therefore provide evidence that subjective caregiver burden is an important risk factor for psychiatric morbidity in carers. Depressive symptoms originate from stress responses and are associated with high levels of psychological distress [4]; however stressors do not cause depressive symptoms directly [19]. They can be conceptualised as the consequences of appraising the caregiving situation as highly stressful whereby high levels of subjective caregiver burden are associated with increased risk of experiencing psychiatric distress [96].

We tested several hypotheses in relation to sources of heterogeneity between studies. Our sensitivity analyses showed that the pooled effect of subjective caregiver burden on depressive symptoms was higher in dementia caregivers compared to those caring for frail older people, or stroke survivors similar to the Pinquart and Sorensen [20] review. Type of care recipient illness therefore was an important source of heterogeneity. Our findings add new evidence that dementia may differentially affect caregiver burden and risk of experiencing depressive symptoms for carers [20]. Carer age, sex and relationship to care recipient on the other hand did not explain heterogeneity between studies. Studies that employed non-probabilistic sampling showed a higher pooled effect estimate compared to those using probabilistic sampling; this indicates that non-probabilistic sampling overestimates the effect of subjective caregiver burden on depressive symptoms.

Although our study is the first comprehensive meta-analysis in the literature, it has several limitations. Our meta-analysis has not been registered online and it was not possible to control for several confounders such as prior history of depression, influence of individual patient behavioural and psychological symptoms [97] and time-varying characteristics of subjective caregiver burden, which may have influenced our results. Studies used different scales to measure subjective caregiver burden and this may have added to heterogeneity. Further longitudinal epidemiological research is warranted to establish significant mediators of the association of subjective burden and depressive symptoms.

Despite limitations the results of our review have significant clinical implications. We have been able to demonstrate that subjective caregiver burden may signal clinical depression in family carers of frail older people. Screening questions by clinicians will be useful in identifying carers at increased risk of psychological distress. Our findings support the use of interventions aimed at alleviating subjective caregiver burden to prevent depressive symptoms and psychiatric morbidity in this population. Interventions for example that target cognitive reappraisals, teach coping strategies and provide emotional support, are effective in reducing caregiver burden [98] and may protect carers’ mental health via reinforcing protective psychological mechanisms [99]. More research is needed in order to strengthen the evidence and understand which factors associated with caregiver burden may be responsive to change by psychological interventions.

## Supporting information

S1 Appendix Syntax used in each database. (DOCX)

S1 Checklist PRISMA checklist for systematic reviews. (DOCX)
