# Supplementary material for: The association between subjective caregiver burden and depressive symptoms in carers of older relatives: A systematic review and meta-analysis
Source: PLoS One. 2019 May 29;14(5):e0217648. doi: 10.1371/journal.pone.0217648 (PMC6541277; doi:10.1371/journal.pone.0217648)
Supplement: S1 Appendix — (DOCX) [file pone.0217648.s001.docx]

PubMed:

(Caregivers[mj] or carer*) and (depression[mj] or (depress* not medline[sb])) and (burden or strain or role overload)

Cinahl:

(MH Caregivers or carer*) and (MH depression or AB depress*) and (AB burden or AB strain or AB role overload)

PsychInfo:

(SU(Caregivers) or AB(carer*)) and (SU(depression)) and (AB(burden) or AB(strain) or AB(role overload))

Scopus

(INDEXTERMS(Caregivers) or TITLE-ABS-KEY(carer*)) and (INDEXTERMS (depression) or TITLE-ABS-KEY(depress*)) and (TITLE-ABS-KEY (burden) or TITLE-ABS-KEY (strain) or TITLE-ABS-KEY (role overload))
